# Mifepristone inhibited the expression of B7-H2, B7-H3, B7-H4 and PD-L2 in adenomyosis

**DOI:** 10.1186/s12958-021-00800-6

**Published:** 2021-07-21

**Authors:** Xiaoyan Qin, Wenjing Sun, Chong Wang, Mingjiang Li, Xingbo Zhao, Changzhong Li, Hui Zhang

**Affiliations:** 1 Department of Obstetrics and Gynaecology, Shandong Provincial Hospital Affiliated to Shandong First Medical University, Jinan, Shandong 250021 People’s Republic of China; 2 Department of Surgery, Shandong Rongjun General Hospital, Jinan, Shandong 250013 People’s Republic of China; 3 Department of Obstetrics and Gynaecology, Shandong University, Jinan, Shandong 250000 People’s Republic of China

**Keywords:** B7-H2, B7-H3, B7-H4, PD-L2, Mifepristone, Adenomyosis

## Abstract

**Background:**

The immune mechanism was shown to be involved in the development of adenomyosis. The aim of the current study was to evaluate the expression of the immune checkpoints B7-H2, B7-H3, B7-H4 and PD-L2 in adenomyosis and to explore the effect of mifepristone on the expression of these immune checkpoints.

**Methods:**

The expression of B7-H2, B7-H3, B7-H4 and PD-L2 in normal endometria and adenomyosis patient samples treated with or without mifepristone was determined by immunohistochemistry analysis.

**Results:**

In adenomyosis patient samples, the expression of B7-H2, B7-H3 and B7-H4 was increased in the eutopic and ectopic endometria compared with normal endometria, both in the proliferative and secretory phases. Moreover, the expression of B7-H2 and B7-H3 was higher in adenomyotic lesions than in the corresponding eutopic endometria, both in the proliferative and secretory phases. The expression of PD-L2 was higher in adenomyotic lesions than in normal endometria in both the proliferative and secretory phases. In the secretory phase but not the proliferative phase, the expression of B7-H4 and PD-L2 in adenomyotic lesions was significantly higher than that in the corresponding eutopic endometria. In normal endometria and eutopic endometria, the expression of B7-H4 was elevated in the proliferative phase compared with that in the secretory phase, while in the ectopic endometria, B7-H4 expression was decreased in the proliferative phase compared with the secretory phase. In addition, the expression of B7-H2, B7-H3, B7-H4 and PD-L2 was significantly decreased in adenomyosis tissues after treatment with mifepristone.

**Conclusions:**

The expression of the immune checkpoint proteins B7-H2, B7-H3, B7-H4 and PD-L2 is upregulated in adenomyosis tissues and is downregulated with mifepristone treatment. The data suggest that B7 immunomodulatory molecules are involved in the pathophysiology of adenomyosis.

## Background

Adenomyosis is a chronic inflammatory disease characterized by the invasion and growth of functional endometrial glands and stroma in the myometrium. Adenomyosis always causes dysmenorrhea, menorrhea and subfertility, which seriously affect the physical and psychological health of women. However, its pathogenesis remains poorly understood [1]. It has been reported that estrogen exposure, adhesion molecules, extracellular matrix metalloproteinase and pro-inflammatory cytokines were involved in pathogenesis of adenomyosis and endometriosis by creating the conditions for differentiation, adhesion, proliferation and survival of ectopic endometrial cells [2–4]. Growing evidence from diverse studies has shown that aberrant immune responses play a vital role in the pathogenesis of adenomyosis [5]. Both systemic and local immune alterations exist in women affected by adenomyosis, with the coexistence of changes in inflammatory and anti-inflammatory signals [5]. This underscores the immune contributions to the disease.

The B7-CD28 family includes immunomodulatory molecules and indispensable factors for complete T cell activation. Recently, the newly identified B7 proteins B7-H2, B7-H3, B7-H4 and programmed death ligand 2 (PD-L2, CD273, B7-DC) have attracted increasing attention. These cosignaling molecules not only provide critical positive signals that stimulate T cell growth, upregulate cytokine production and promote T cell differentiation but also contribute key negative signals to limit, terminate and/or attenuate T cell responses [6, 7]. Aberrant expression of these B7 family members is associated with the emergence of T cell exhaustion in many disorders, including cancers, pregnancy, and autoimmune diseases [8]. Therefore, we wondered whether altered expression of these B7 family members could be observed in adenomyosis.

To date, hysterectomy is still the most common and effective treatment for adenomyosis, as it nearly ensures cure. However, treatment is a challenge as many patients desire uterus preservation [9]. For patients for whom hysterectomy is contraindicated, drug therapy, including oral contraceptives, progestogen, mifepristone, danazol and gonadotropin-releasing hormone analogs, are important therapeutic approaches. It has been shown that mifepristone can inhibit endometrial proliferation or suppress adenomyotic lesions, resulting in inhibition of prostaglandin production and endometrial atrophy in animal models [10]. It has been demonstrated that treatment with 50 mg of mifepristone daily leads to improved pain and regression of adenomyosis [11].

Therefore, in the current study, we determined the expression and localization of four B7 molecules, B7-H2, B7-H3, B7-H4 and PD-L2, in adenomyosis patient samples and normal control samples. In addition, we also investigated the effect of mifepristone on the expression of these four B7 molecules in adenomyosis.

## Methods

### Collection of tissues

The study participants were enrolled at the Department of Obstetrics and Gynaecology in the Provincial Hospital affiliated to Shandong First Medical University from January 2016 to March 2018. Adenomyotic lesions and the corresponding eutopic endometria were obtained from patients with adenomyosis undergoing hysterectomy. The drug-untreated adenomyosis sample group comprised 58 patients with adenomyosis (proliferative phase: *n* = 35; secretory phase: *n* = 23) with no use of any hormone therapy within at least 6 months before surgery. The mifepristone-treated adenomyosis sample group consisted of 11 patients with adenomyosis treated with mifepristone at a dose of 12.5 mg daily for 3 months. Seventy-four samples of normal endometrium collected from hysterectomy specimens and checked for leiomyoma pathology (proliferative phase: *n* = 47; secretory phase: *n* = 27) were included as controls. Participants in the control group had no evidence of adenomyosis in the histopathological examination of their hysterectomy specimens and had no visible pelvic inflammation or endometriosis at the time of hysterectomy. The characteristics of the patients in each group are shown in Table 1. Ectopic and eutopic endometria of adenomyosis patients were collected during the surgery. Adenomyosis was confirmed by histological examination. Written informed consent was obtained from all participants prior to the biopsy procedure. This study was approved by the Institutional Review Board of Shandong First Medical University.

**Table 1:** The characteristics of participants in each group

	N	Age ranges (yrs)	Mean (yrs)	STD
Control group				
Proliferative stage	47	38–52	46.9149	3.0132
Secretory stage	27	39–52	46.5556	3.0298
Adenomyosis				
Proliferative stage	35	32–52	44.6286	4.2294
Secretory stage	23	40–52	45.6857	3.2813
Adenomyosis treated with mifepristone	11	39–52	45.8182	3.8424

*Abreviations*: *N* number, *yrs* years, *STD* standard deviation

### Immunohistochemistry analysis

The tissue processing and staining procedure was described in detail in our previous study [12]. Immunohistochemical staining was performed using polyclonal antibody rabbit anti-human ICOSL (B7-H2) (1:100, ab233151, Abcam, Cambridge, UK), monoclonal antibody mouse anti-human CD276 (B7-H3) (1:200, ab105922, Abcam), monoclonal antibody rabbit anti-human VTCN1 (B7-H4) (1:300, ab209242, Abcam) and polyclonal antibody rabbit anti-human PD-L2 (1:100, ab244332, Abcam). Goat anti-rabbit and goat anti-mouse HRP-conjugated secondary antibodies and diaminobenzidine (DAB) staining kits were obtained from ZSGB-BIO (Beijing, China).

The sections were viewed under a Leica DM4000B microscope (Leica, Wetzlar, Germany), and photographs were taken using the IM50 image analysis system (Leica). Immunohistochemical staining was evaluated by a semiquantitative immunoscore, which was calculated as the product of the quantity score and staining intensity. The quantity score (Pi), i.e., the percentage of positively stained glandular epithelial cells, was estimated from 100 cells counted in four randomly chosen views. The staining intensity (I) of the glandular epithelial cells was estimated as follows: 0: negative; 1: weak staining; 2: moderate staining; and 3: strong staining. The H-score was calculated as follows: Pi* (I + 1). Two sections of each sample were assessed by two investigators blinded to any pathological or clinical data about the tissues. The average score of the two investigators was used.

### Statistical analysis

Statistical analysis of data was performed by analysis of variance using SPSS 19.0 (SPSS Inc., Chicago, IL). Data are presented as means ± SD. Differences between two groups were determined by a two-tailed Student’s t-test. *P* < 0.05 was considered to be statistically significant.

## Results

### Overexpression of B7-H2 in adenomyotic eutopic and ectopic endometria

**Fig. 1:**
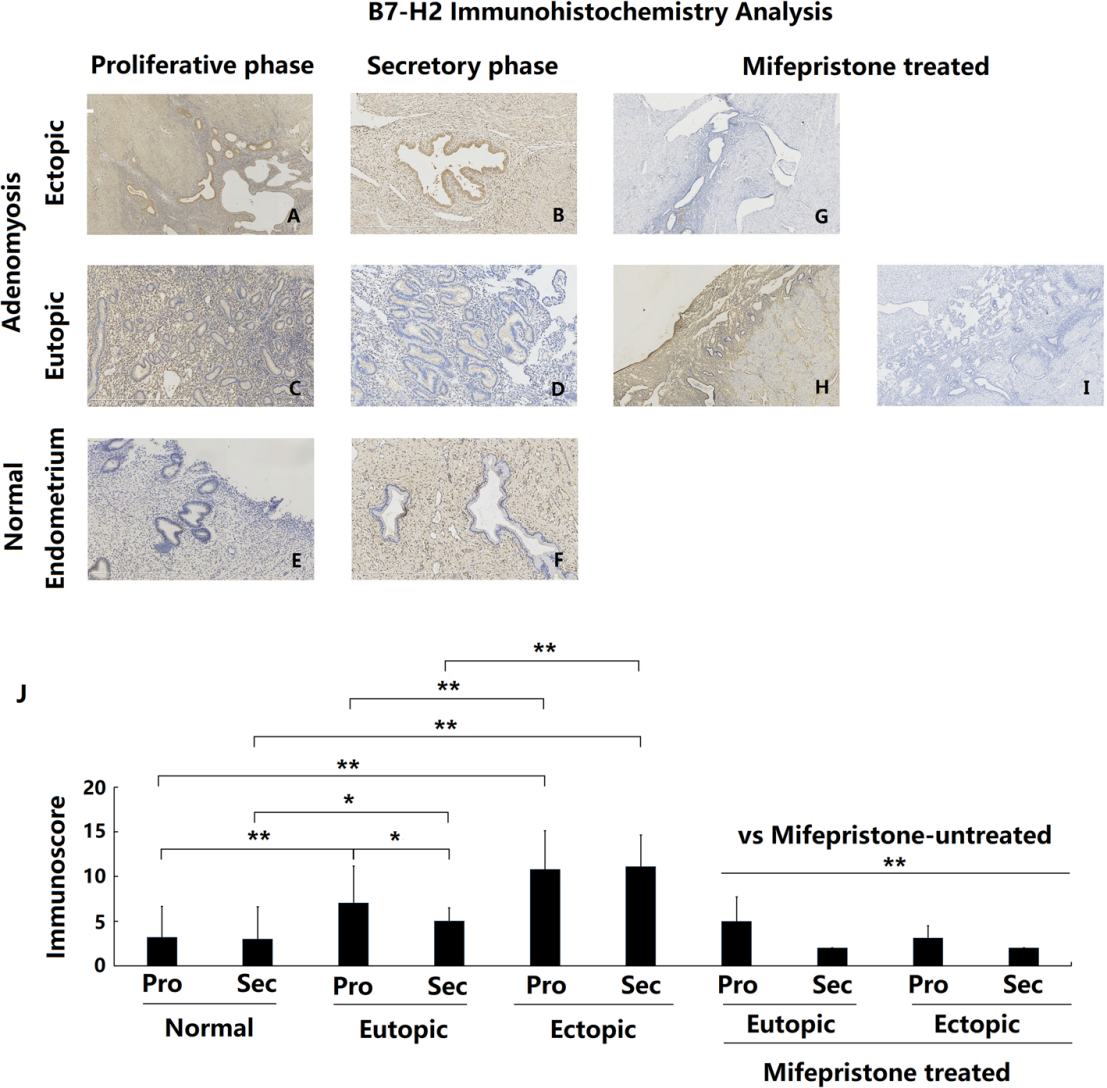
Immunoexpression and comparison of B7-H2 in normal, eutopic and ectopic endometrium of adenomyosis treated with and without mifepristone. **a** Ectopic endometrium of proliferative phase in patient with untreated adenomyosis (*n* = 35); **b** Ectopic endometrium of secretory phase in patient with untreated adenomyosis (*n* = 23); **c** Eutopic endometrium of proliferative phase in patient with untreated adenomyosis (*n* = 35); **d** Eutopic endometrium of secretory phase in patient with untreated adenomyosis (*n* = 23); **e** Normal endometrium of proliferative phase in patients without adenomyosis (*n* = 47); **f** Normal endometrium of secretory phase in patients without adenomyosis (*n* = 27); **g** Ectopic endometrium in patient with mifepristone-treated adenomyosis (*n* = 11); **h** and **i** Eutopic endometrium in patient with mifepristone-treated adenomyosis (*n* = 11); **j** Immunoscore comparation of B7-H2 between each groups,* *P* < 0.05, ** *P* < 0.01. a- i magnification: × 100

B7-H2 protein was expressed both in glandular and stromal cells in the endometrial tissues from adenomyosis patients, mainly in the glandular epithelial cells. Positive expression was mainly detected in the cell membrane and cytoplasm but not in the nucleus. The expression of B7-H2 protein in the endometrial tissues of the control group samples was extremely faint and almost absent from both glandular epithelial and stromal cells (Fig. 1e and f). In contrast, B7-H2 protein was moderately expressed in some epithelial and stromal cells in the eutopic endometrium from adenomyosis patients (ADE-EU) (Fig. 1c and d) and intensely expressed in the ectopic endometrium from adenomyosis patients (ADE-EC) (Fig. 1a and b). Compared with the control group, ADE-EU and ADE-EC showed significantly increased expression of B7-H2, both in the proliferative and secretory endometrium phases. (Fig. 1j ADE-EU vs control: *P* (proliferative stage) < 0.001; *P* (secretory stage) = 0.015; ADE-EC vs control (proliferative and secretion stage): both *P* < 0.001). Moreover, the expression of B7-H2 in ADE-EC was significantly higher than that in ADE-EU in the same menstrual cycle phase (Fig. 1j ADE-EC vs ADE-EU (proliferative and secretion stage): both *P* < 0.001).

During the menstrual cycle, the expression of B7-H2 in the ADE-EU group was significantly stronger in the proliferative stage than in the secretory stage (*P* = 0.03), while no significant difference was noted in the ADE-EC group (*P* = 0.78) or in the control group (*P* = 0.82).

### Overexpression of B7-H3 in adenomyotic eutopic and ectopic endometria

**Fig. 2:**
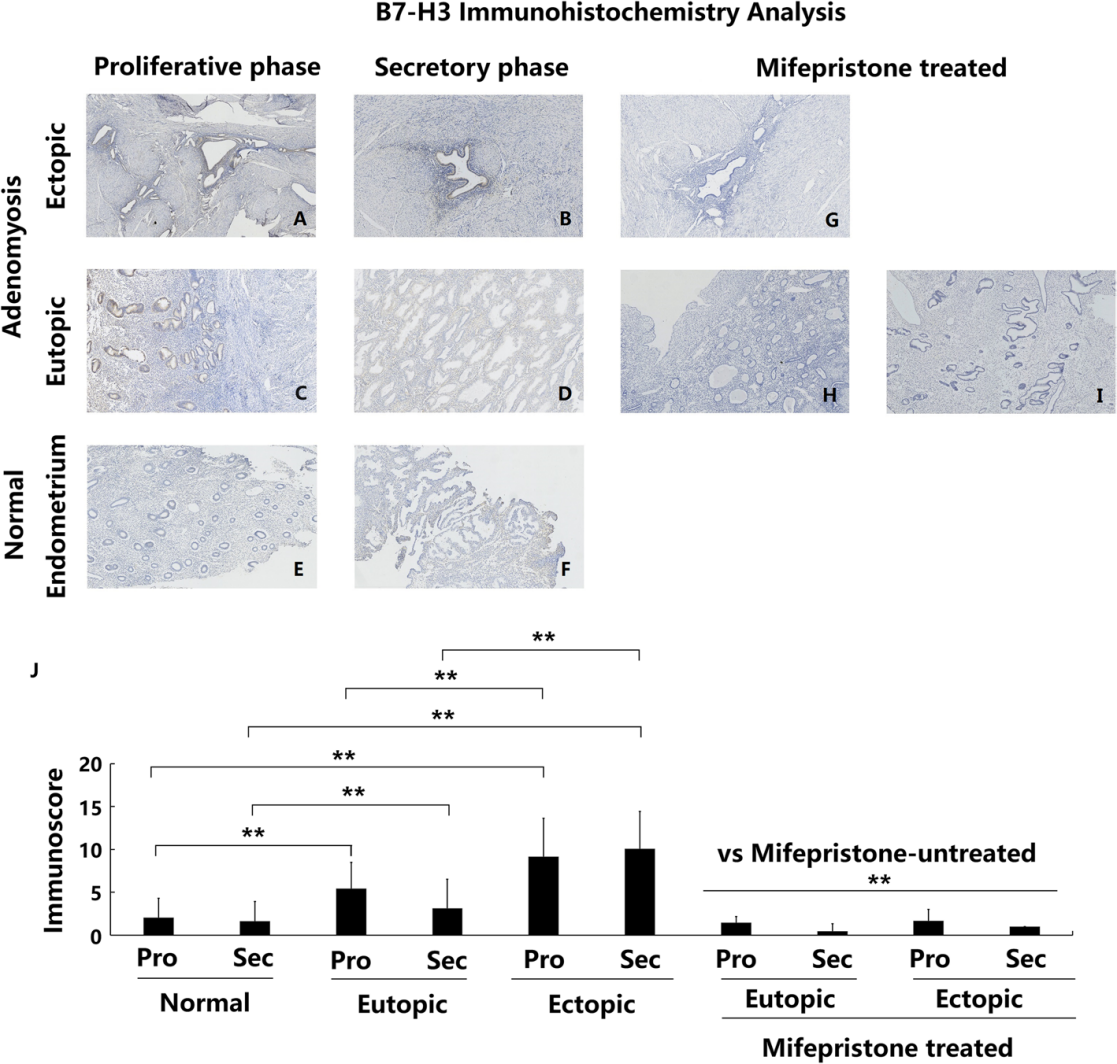
Immunoexpression and comparison of B7-H3 in normal, eutopic and ectopic endometrium of adenomyosis treated with and without mifepristone. **a** Ectopic endometrium of proliferative phase in patient with untreated adenomyosis (*n* = 35); **b** Ectopic endometrium of secretory phase in patient with untreated adenomyosis (*n* = 23); **c** Eutopic endometrium of proliferative phase in patient with untreated adenomyosis (*n* = 35); **d** Eutopic endometrium of secretory phase in patient with untreated adenomyosis (*n* = 23); **e** Normal endometrium of proliferative phase in patients without adenomyosis (*n* = 47); **f** Normal endometrium of secretory phase in patients without adenomyosis (*n* = 27); **g** Ectopic endometrium in patient with mifepristone-treated adenomyosis (*n* = 11); **h** and **i** eutopic endometrium in patient with mifepristone-treated adenomyosis (*n* = 11); **j** Immunoscore comparation of B7-H3 between each groups, * *P* < 0.05, ** *P* < 0.01. a- i magnification: × 100;

B7-H3 protein was mainly expressed in the glandular epithelium instead of the stromal cells in endometrial tissues. Positive expression was mainly located in the cell membrane and cytoplasm. The expression of B7-H3 protein in the endometrial tissues of the control group was weak, and almost no expression was observed in either the glandular or stromal cells (Fig. 2e and f). However, B7-H3 was moderately expressed in the glandular epithelium of ADE-EU samples (Fig. 2c and d), and intensely expressed in ADE-EC samples (Fig. 2a and b). Compared with the control group, ADE-EU tissues showed significantly increased B7-H3 expression in both the proliferative and secretory phases (Fig. 2j ADE-EU vs control: *P* (proliferative phase) < 0.001; *P* (secretory phase) = 0.002). Moreover, ADE-EC tissues showed significantly higher expression of B7-H3 than the corresponding ADE-EU tissue at the same menstrual cycle phase (Fig. 2j ADE-EC vs ADE-EU (proliferative and secretion stage): both *P* < 0.001).

### Overexpression of B7-H4 in adenomyotic eutopic and ectopic endometria

In the endometrium of patients with or without adenomyosis, immunostaining for B7-H4 revealed similar characteristics. Positive staining was mainly observed in the glandular epithelial cells, with almost no staining in the stroma. The positive B7-H4 immunostaining was located mainly in the cell membrane and cytoplasm.

**Fig. 3:**
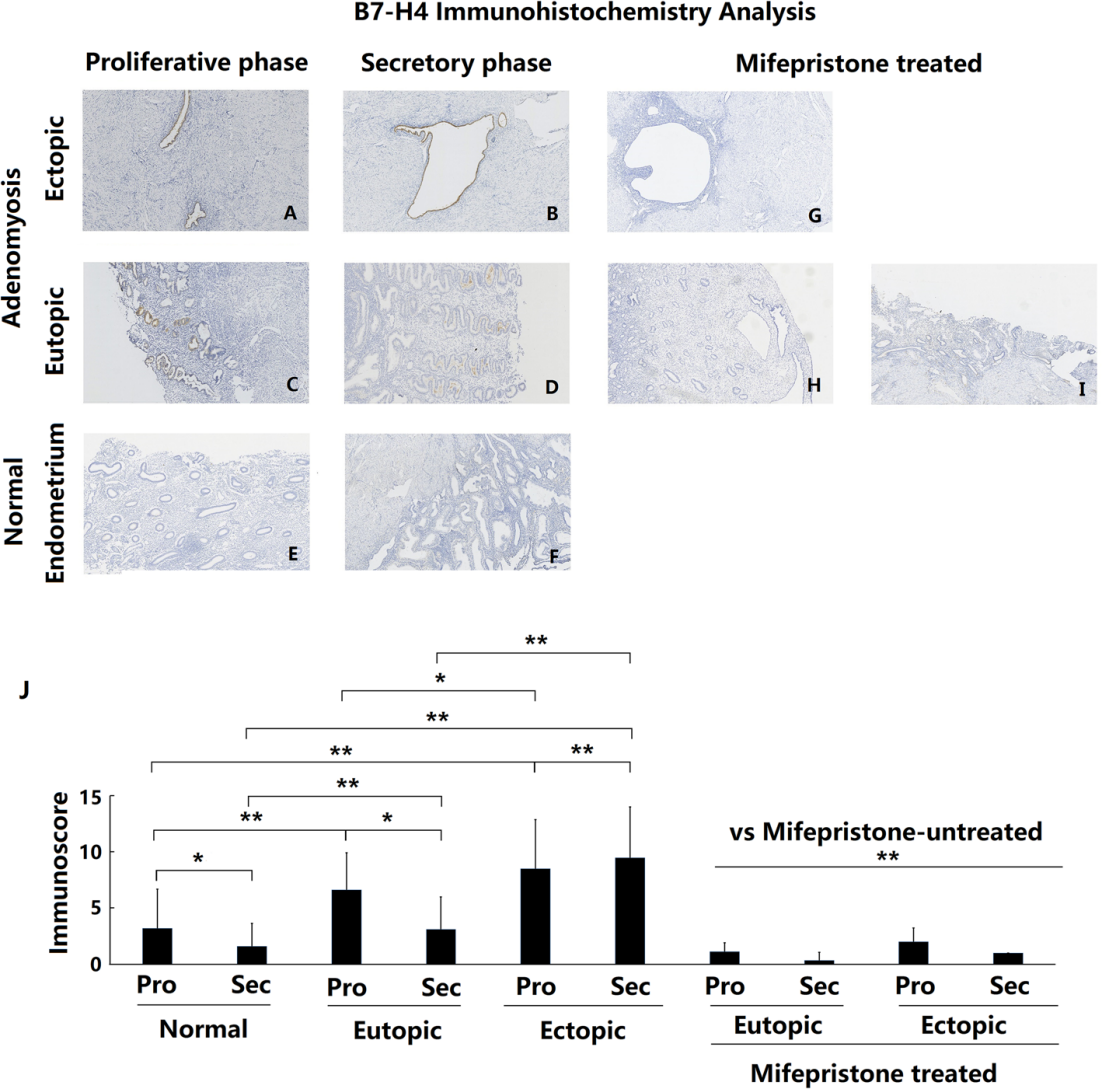
Immunoexpression and comparison of B7-H4 in normal, eutopic and ectopic endometrium of adenomyosis treated with and without mifepristone. **a** Ectopic endometrium of proliferative phase in patient with untreated adenomyosis (*n* = 35); **b** Ectopic endometrium of secretory phase in patient with untreated adenomyosis (*n* = 23); **c** Eutopic endometrium of proliferative phase in patient with untreated adenomyosis (*n* = 35); **d** Eutopic endometrium of secretory phase in patient with untreated adenomyosis (*n* = 23); **e** Normal endometrium of proliferative phase in patients without adenomyosis (*n* = 47); **f** Normal endometrium of secretory phase in patients without adenomyosis (*n* = 27); **g** Ectopic endometrium in patient with mifepristone-treated adenomyosis (*n* = 11); **h** and **i** Eutopic endometrium in patient with mifepristone-treated adenomyosis (*n* = 11); **j** Immunoscore comparation of B7-H4 between each groups,* *P* < 0.05, ** *P* < 0.01. a- i magnification: × 100

In the control group, the B7-H4 expression levels in the endometria were extremely low, with immunostaining almost absent (Fig. 3e and f). In the adenomyosis group, ADE-EU tissues had moderate B7-H4 immunostaining in the glandular epithelium cells (Fig. 3c and d). ADE-EC tissues had strong B7-H4 immunostaining intensity in almost all glandular epithelial cells (Fig. 3a and b). Compared with the control group, ADE-EU and ADE-EC tissues showed significantly higher expression of B7-H4 both in the proliferative and secretory phases (Fig. 3j *P* < 0.001). ADE-ECs in the secretory phase showed significantly higher levels of B7-H4 expression than the corresponding ADE-EUs in the same menstrual cycle phase (Fig. 3j *P* < 0.001). However, no significant difference in B7-H4 expression was shown between ADE-EU and ADE-EC tissues in the proliferative phase (Fig. 3j *P* = 0.06).

In both the ADE-EU and control groups, the expression of B7-H4 was higher in the proliferative phase than in the secretory phase (Fig. 3j control group: *P* = 0.01, ADE-EU:*P* = 0.03). In contrast, the expression in ADE-ECs was lower in the proliferative phase than in the secretory phase (Fig. 3b). J *P* < 0.001).

### Overexpression of PD-L2 in ectopic endometria of patients with adenomyosis

**Fig. 4:**
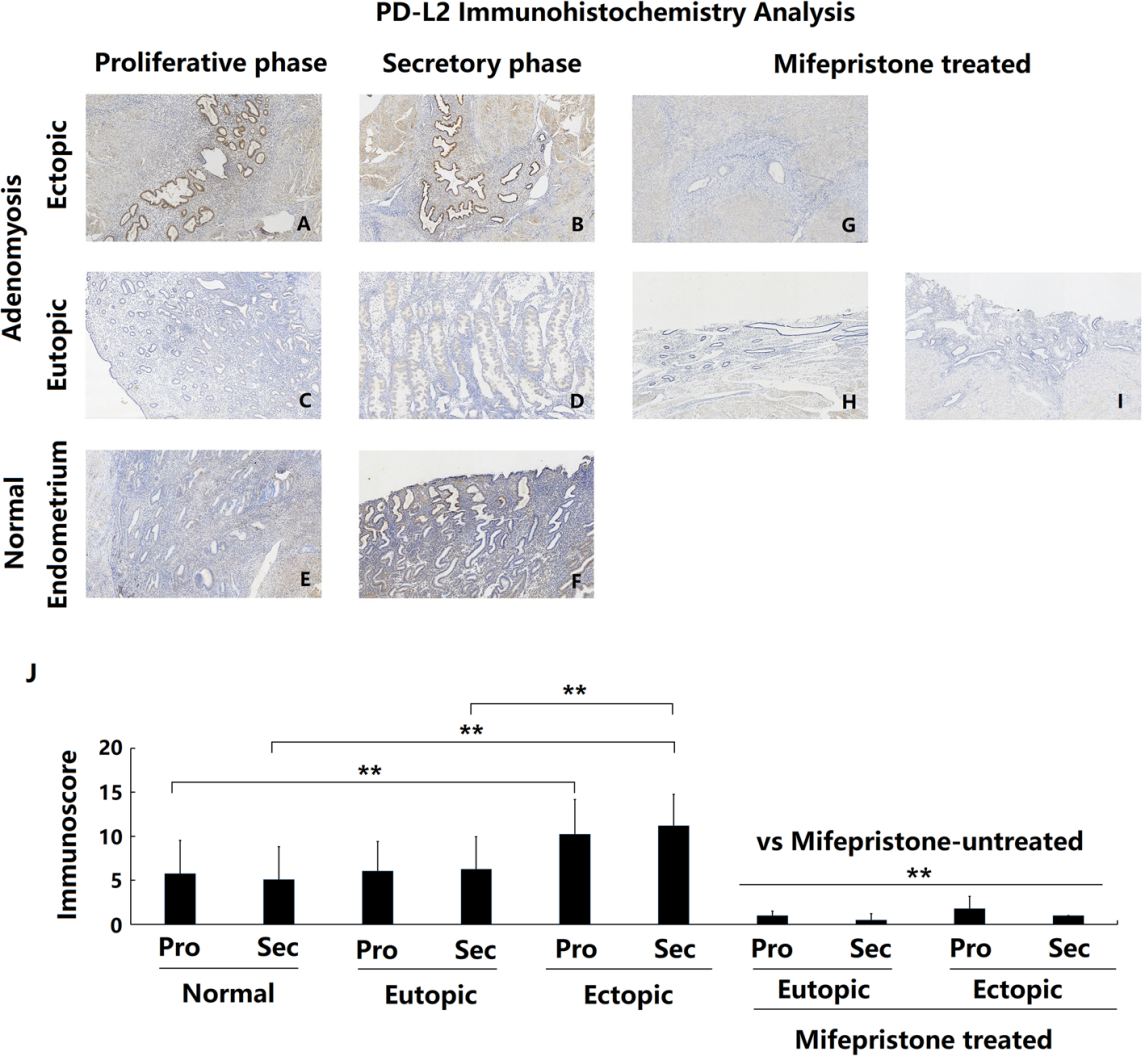
Immunoexpression and comparison of PD-L2 in normal, eutopic and ectopic endometrium of adenomyosis treated with and without mifepristone. **a** Ectopic endometrium of proliferative phase in patient with untreated adenomyosis (*n* = 35); **b** Ectopic endometrium of secretory phase in patient with untreated adenomyosis (*n* = 23); **c** Eutopic endometrium of proliferative phase in patient with untreated adenomyosis (*n* = 35); **d** Eutopic endometrium of secretory phase in patient with untreated adenomyosis (*n* = 23); **e** Normal endometrium of proliferative phase in patients without adenomyosis (*n* = 47); **f** Normal endometrium of secretory phase in patients without adenomyosis (*n* = 27); **g** Ectopic endometrium in patient with mifepristone-treated adenomyosis (*n* = 11); **h** and **i**. Eutopic endometrium in patient with mifepristone-treated adenomyosis (*n* = 11); **j** Immunoscore comparation of PD-L2 between each groups,* *P* < 0.05, ** *P* < 0.01. a- i magnification: × 100;

The expression of PD-L2 protein in the control and adenomyotic endometrium was determined using immunohistochemical analysis. Figure 4 shows that PD-L2 was not expressed or only weakly expressed in the endometrium of both the control group (Fig. 4e and f) and the ADE-EU group (Fig. 4c and d). Notably, PD-L2 was expressed at significantly higher levels in the endometrium of ADE-EC samples (Fig. 4a and b) compared with the control group, both in the proliferative and secretory phases (Fig. 4j both *P* < 0.01). Moreover, ADE-EC tissues showed higher PD-L2 expression than ADE-EU tissues in the secretory but not the proliferative phase. Nevertheless, there was no significant difference in PD-L2 expression between the ADE-EU group and the control group (Fig. 4j). The data also showed that in the ectopic endometria of adenomyosis samples, PD-L2 was primarily expressed in the glandular epithelial cells. During the menstrual cycle, no periodic changes in endometrial PD-L2 expression were found in either the adenomyosis group or the control group.

**Fig. 5:**
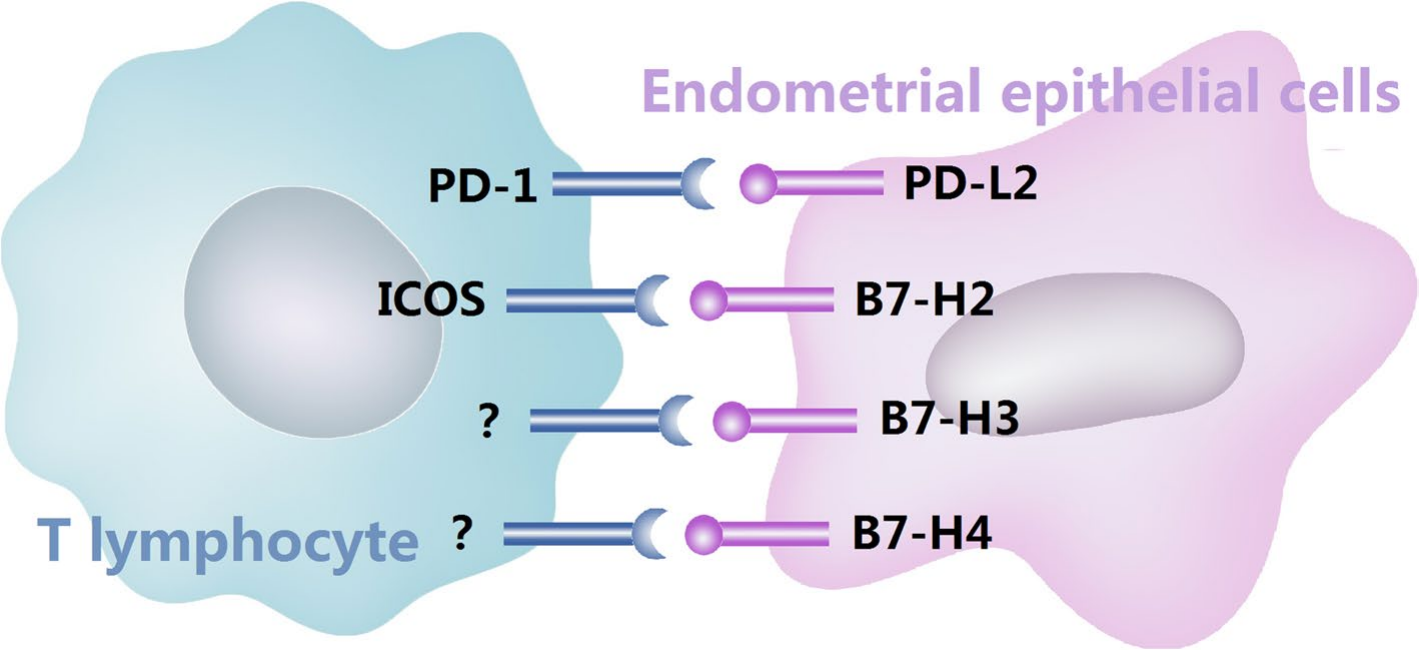
Schematic representation of B7-H2, B7-H3, B7-H4 and PD-L2 with their respective receptor

### Decreased expression of B7-H2, B7-H3, B7-H4 and PD-L2 in patients with adenomyosis after mifepristone treatment

Immunohistochemical staining was used to detect and compare the changes in B7-H2, B7-H3, B7-H4 and PD-L2 expression in the eutopic and ectopic endometrium of patients with adenomyosis with and without mifepristone treatment. The results showed that the eutopic and ectopic endometrium of adenomyosis patients treated with mifepristone showed significantly lower expression of B7-H2, B7-H3, B7-H4 and PD-L2 protein compared with that of adenomyosis patients without mifepristone treatment, both in the proliferative phase and in the secretory phase (Figs. 1, 2, 3 and 4g-j all *P* < 0.01).

## Discussion

The activation of T lymphocytes plays an essential role in the process of immunity. This activation requires two signals simultaneously [13]. 1) The first stimulus signal is provided by T cell receptor upon recognition by the MHC-antigen complex; 2) the key second signal is delivered by the binding of T cells to comodulatory molecules expressed on antigen-presenting cells (APCs); these molecules include those in the B7 family. In recent years, a breakthrough was made in the targeting of the three main members of the B7 family, CTLA-4, PD-1 and PD-L1 (B7-H1), in the immune checkpoint blockade treatment of cancer [13]. The B7 family proteins are the most important immunomodulatory molecules. Since there is little knowledge of the correlation between the B7 family and adenomyosis, our study explored the expression of B7-H2, B7-H3, B7-H4 and PD-L2 in adenomyosis patients with and without mifepristone treatment.

B7-H2 is expressed on professional APCs and binds to the inducible costimulatory molecule (ICOS) expressed on T cells. The ICOS/B7-H2 signal is involved in several aspects of the T-cell response. This engagement plays an essential role in the differentiation of CD4^+^ T cells into effector subsets, including Th1, Th2, Th17 and regulatory T cells (Tregs) [14, 15]. In acute myelocytic leukemia (AML), strongly-activating B7-H2 exhibits an inhibitory function by which enabled AML cells to facilitate immune escape [16]. Recently, overexpression of B7-H2 was found in a variety of solid cancers and was found to maintain the immunosuppressive Treg subset, which is associated with tumor progression and poor overall survival [17]. Th17/Treg imbalance has been shown to be present in adenomyosis [18]. Considering the effect of B7-H2 on the differentiation of Th17 cells and Tregs from CD4^+^ T cells, it is reasonable to postulate that B7-H2 is involved in the pathogenesis of adenomyosis. Further studies are needed to elucidate the definitive mechanisms involved. Furthermore, the engagement of B7-H2 with ICOS can stimulate IFN-gamma, IL-4, IL-5 and IL-10 production by T cells; IL-10 is most effectively induced [7, 19, 20]. Moreover, ICOS stabilizes IL-10R expression on T cells, rendering them sensitive to IL-10 [21]. This may add to the interpretation of our previous findings that the expression of IL-10 [22] and IL-10R [23] is upregulated in the eutopic and ectopic endometrium of adenomyosis patients. B7-H2 plays a primary regulatory role and promotes the Th2 immune response. In B7-H2-deficient mice, the production of Th2 cytokines such as IL-4 and IL-10 by primed T cells is reduced [24]. Shifts towards the Th2 immune response have been found to be involved in endometriosis, with a relative predominance of IL-4 and IL-10 [25]. In this study, we found that the endometrial expression of B7-H2 was higher in patients with adenomyosis than in the control group. Moreover, the intensity of B7-H2 expression was substantially increased in the adenomyotic ectopic endometrium compared with that in the adenomyotic eutopic endometrium. This finding implies that aberrant expression of B7-H2 is involved in the pathogenesis of adenomyosis.

B7-H3 was initially thought to costimulate the immune response, but recent studies have shown that it is predominantly a T-cell coinhibitory molecule that contributes to immune evasion [26, 27]. B7-H3 is widely expressed in both lymphoid and nonlymphoid organs at the RNA level, but the expression of B7-H3 protein is more restricted to cell types such as activated dendritic cells, monocytes, T cells, B cells, and NK cells. Aberrant expression of B7-H3 has been shown to be associated with poor outcome in various human malignancies [28] and autoimmune diseases [29]. Overexpression of B7-H3 was displayed in 60–93% of tumor tissues in the vast majority of cancer types via immunohistochemical assays, while very limited expression was seen in normal healthy tissue [28]. This was consistent with what we observed in this study: only weak or absent expression of B7-H3 was observed in the normal endometrium; however, B7-H3 immunostaining intensity was increased in adenomyosis patient endometrium. This finding implied that the overexpression of B7-H3 might participate in the genesis of adenomyosis. Diverse studies have demonstrated that upregulation of B7-H3 expression is associated with impaired T-cell stimulation [30, 31], suppressed NK-mediated cell lysis [32], increased IL-10 secretion [33], and modulation of the Jak/Stat pathway [34], which contributes to tumor immune suppression and evasion. These results showed that B7-H3 was involved in tumor progression by acting as a negative regulator of T cells and facilitating tumor immune evasion. Similar to in tumors, B7-H3 also displays coinhibitory properties in some immune diseases. Independent studies utilizing either protein blockade or gene-knockout mice have reported that B7-H3 ameliorates graft-versus-host disease, prolongs cardiac allograft survival, reduces airway hypersensitivity, and delays experimental autoimmune encephalomyelitis onset, in particular by dampening the Th1 response [35–37]. These examples provide further evidence for the coinhibitory properties of B7-H3. Our study found that B7-H3 was overexpressed in adenomyotic endometria compared with the control endometria. Moreover, the adenomyotic ectopic endometria expressed even higher B7-H3 levels in comparison with those of the eutopic tissues. We postulate that the immunologic function of B7-H3 in adenomyosis is similar to that in malignancies and autoimmune diseases and acts as a coinhibitory immunomodulator. The overexpression of B7-H3 enables the endometrium to create an immunosuppressive microenvironment to facilitate the eutopic and ectopic endometrium to escape host immunosurveillance before infiltrating and after infiltrating into the myometrium, thus leading to the origination and progression of adenomyosis.

B7-H4 is a vital B7 ligand that acts as a negative regulator of the T cell-mediated immune response. B7-H4 mRNA is widely distributed in human peripheral tissues. However, B7-H4 protein expression is more restricted in most normal tissues and its expression can be induced on APCs after in vitro stimulation [38]. Recent studies found a negative immunomodulatory role of B7-H4 in a wide range of tumors [39], autoimmune diseases [40], viral infections [41] and transplantation rejection occurrences [42]. In the endometrium, the expression of B7-H4 was estimated in Miyatake T’s study [43]. They showed that the staining of B7-H4 is faint or moderate in the apex of the cytoplasmic membrane in normal or hyperplastic endometrium but that it is strong in the circumferential membrane and cytoplasm in most endometrioid carcinomas [43]. A significant inverse correlation has been observed between the high expression of B7-H4 in the majority of endometrioid carcinomas and the number of tumor infiltrating T cells, particularly the number of tumor-associated CD3^+^ and CD8^+^ lymphocytes [43]. High expression of B7-H4 in the tumor microenvironment exerts negative immunomodulatory effects through several pathways, including arresting the cell cycle at the G0/G1 stage, promoting T cell apoptosis, inhibiting T cell growth, cytokine secretion and development of cytotoxicity [38], thereby affecting the biological behavior of tumor cells, assisting tumor immune escape, and leading to worse patient prognosis [44]. Similarly, our results showed that the endometrial expression of B7-H4 protein in the control group was extremely weak and almost absent. The expression of B7-H4 in the eutopic and ectopic endometrium of adenomyosis patients was significantly higher than that of the control endometrium. We hypothesize that overexpressed B7-H4 in adenomyosis participates in the formation of an immunotolerant environment of the uterus by negatively regulating T cell proliferation, facilitating immune evasion of ectopic endometrial lesions, preventing the effective elimination of lesions and leading to the development of uterine adenomyosis.

PD-L2, one of the two receptors for PD-1, plays crucial roles in the immune checkpoint pathways responsible for the suppression of T-cell activation [45]. PD-L2 expression can be induced on a diverse variety of other immune cells and nonimmune cells depending on microenvironmental stimuli [45]. PD-L2 was shown to be moderately or strongly expressed in most tumor cells, to interact with PD-1 and to dramatically inhibit TCR-mediated proliferation, CD4^+^ T cell cytokine production and T-cell cytolysis [46]. Via utilization of immune checkpoint molecules, tumor cells exert immunomodulatory functions in the tumor microenvironment and escape host immune surveillance. In this study, we found a higher level of PD-L2 expression in ectopic adenomyotic tissue than in normal endometrial tissues and eutopic adenomyotic tissues. In endometrial tissues, PD-L2 expression was present in 47% of 15 samples of normal endometria and in 40% of 30 samples of endometrial cancer [47]. Expression of the PD-1/PD-L1/PD-L2 axis is associated with moderately and poorly differentiated endometrial cancer and type II endometrial cancer, in which higher expression of PD-1, PD-L1 and PD-L2 may cause immunosuppression, which favors tumor growth and negatively affects patient survival [47]. Similarly, our results indicate that the abnormally increased expression of PD-L2 in adenomyosis may repress T-cell activation and alter the immune microenvironment of the ectopic endometrium. This may enable ectopic endometrial cells to evade normal immunological surveillance and initiate adenomyosis.

Compared with normal endomatria, the adenomyotic lession showed increased expression of B7-H3, B7-H4 and PD-L2. This was consistent with what was observed in endometrial cancer that the endometrial cancer showed high expression of B7-H3 [48], B7-H4 [43, 47] and PD-L2 [47]. Although adenomyosis is a type of benign gynecologic disease, its biological behavior characterized by the presence of ectopic endometrial glands and stroma within the myometrium is similar to that of endometrial cancer [49, 50]. Coexistence of uterine adenomyosis is revealed with an incidence of 22.6% (95% CI 12.7–37.1%) in postoperative pathological examinations of endometrial cancer patients [51]. While the correlation between adenomyosis and endometrial cancer is unclear. Increasing evidence suggests that these two diseases share several altered molecular pathways leading to increased angiogenesis, abnormal tissue growth and invasion [52]. In addition, our finding showed that these two diseases might share an overall immune inhibitory local microenvironment created by upregulated expression of B7-H3, B7-H4 and PD-L2.

In this study, B7-H4 expression showed cyclic variation in control endometria and adenomyotic eutopic, with elevated expression in the proliferative phase. This suggests that B7-H4 expression may be regulated by steroid hormones in the normal endometrium. Consistent with this, Papenfuss TL’s results [53] showed that estriol can upregulate the expression of B7-H4 on the surface of dendritic cells, indicating that estrogen can upregulate the expression of B7-H4. Furthermore, the cyclic change in B7-H4 expression was altered in the ectopic endometria of patients with adenomyosis, with lowered expression of B7-H4 in the proliferative phase. These data suggest that aberrant hormonal sensitivity of B7-H4 in adenomyotic foci may participate in the establishment of this disease.

Compared with untreated adenomyosis, downregulated B7-H2, B7-H3, B7-H4 and PD-L2 expression was observed in adenomyosis patients treated with mifepristone, both in the eutopic and ectopic endometria. To the best of our knowledge, this is the first study to address the effect of mifepristone on B7-H2, B7-H3, B7-H4 and PD-L2 expression. Mifepristone (RU486) is an antiprogestin with a high affinity for progesterone and glucocorticoid receptors. Studies of pregnant women showed that mifepristone can alter the endometrial immune balance and result in implantation failure. It has been shown that mifepristone exerts its effect by shifting immunological elements by enhancing the expression of cytotoxic lymphocytes [54], increasing the cytotoxicity of peripheral blood NK cells [55] and uterine NK cells [56], and enhancing antigen-specific CD4^+^ and CD8^+^ T cell inflammatory cytokines (IFN-γ) and cytotoxic molecule release (granzyme B) [57]. Mifepristone regulates Treg function mediated by dendritic cells by inhibiting the expression of TGF-β [58]. The results of our current study imply that mifepristone may play a therapeutic role in adenomyosis by inhibiting the expression of these four immunomodulatory molecules, improving the immune microenvironment status of the uterus and effectively inhibiting or clearing ectopic endometrial cells. However, further studies are needed to explore the signaling mechanisms underlying our findings.

## Conclusions

Overall, the results described in this study are the first to demonstrate the expression of B7-H2, B7-H3, B7-H4 and PD-L2 by immunostaining in patients with adenomyosis. The differential expression of these proteins in the endometrium with or without adenomyosis suggests that the altered immunomodulatory molecules participate in the pathogenesis of adenomyosis. We postulate that these powerful immunomodulators are critical for providing the eutopic and ectopic endometrium with a suitable immunological environment, leading to ineffective elimination of abnormal eutopic and ectopic endometria and resulting in the initiation and maintenance of adenomyosis (Fig. 5). These findings enrich our comprehension of the local immune status involved in the pathogenesis of adenomyosis and provide potential targets for immunotherapy. The results of this study further demonstrated the possible mechanism of mifepristone treatment on adenomyosis and may provide theoretical data for future studies of adenomyosis treatment. However, the primary limitation of the present study is that we only examined the expression and location of B7-H2, B7-H3, B7-H4 and PD-L2 in adenomyosis patients treated with or without mifepristone by immunohistochemistry. In our future study, we will further investigate the involvement of these B7 family immunomodulators in adenomyosis using multiple experimental techniques.

## Data Availability

The datasets used and analyzed during the present study are available from the corresponding author on reasonable request.
