# Correction: Mifepristone inhibited the expression of B7-H2, B7-H3, B7-H4 and PD-L2 in adenomyosis

**DOI:** 10.1186/s12958-023-01158-7

**Published:** 2023-10-31

**Authors:** Xiaoyan Qin, Wenjing Sun, Chong Wang, Mingjiang Li, Xingbo Zhao, Changzhong Li, Hui Zhang

**Affiliations:** 1 Department of Obstetrics and Gynaecology, Shandong Provincial Hospital Affiliated to Shandong First Medical University, Jinan, Shandong 250021 People’s Republic of China; 2 Department of Surgery, Shandong Rongjun General Hospital, Jinan, Shandong 250013 People’s Republic of China; 3 Department of Obstetrics and Gynaecology, Shandong University, Jinan, Shandong 250000 People’s Republic of China

**Correction: Reprod Biol Endocrinol 19, 114 (2021)**

**https://doi.org/10.1186/s12958-021-00800-6**

Following publication of the original article [1], the authors identified an error in Fig. 2. The correct figure is given below.

**Fig. 2:**
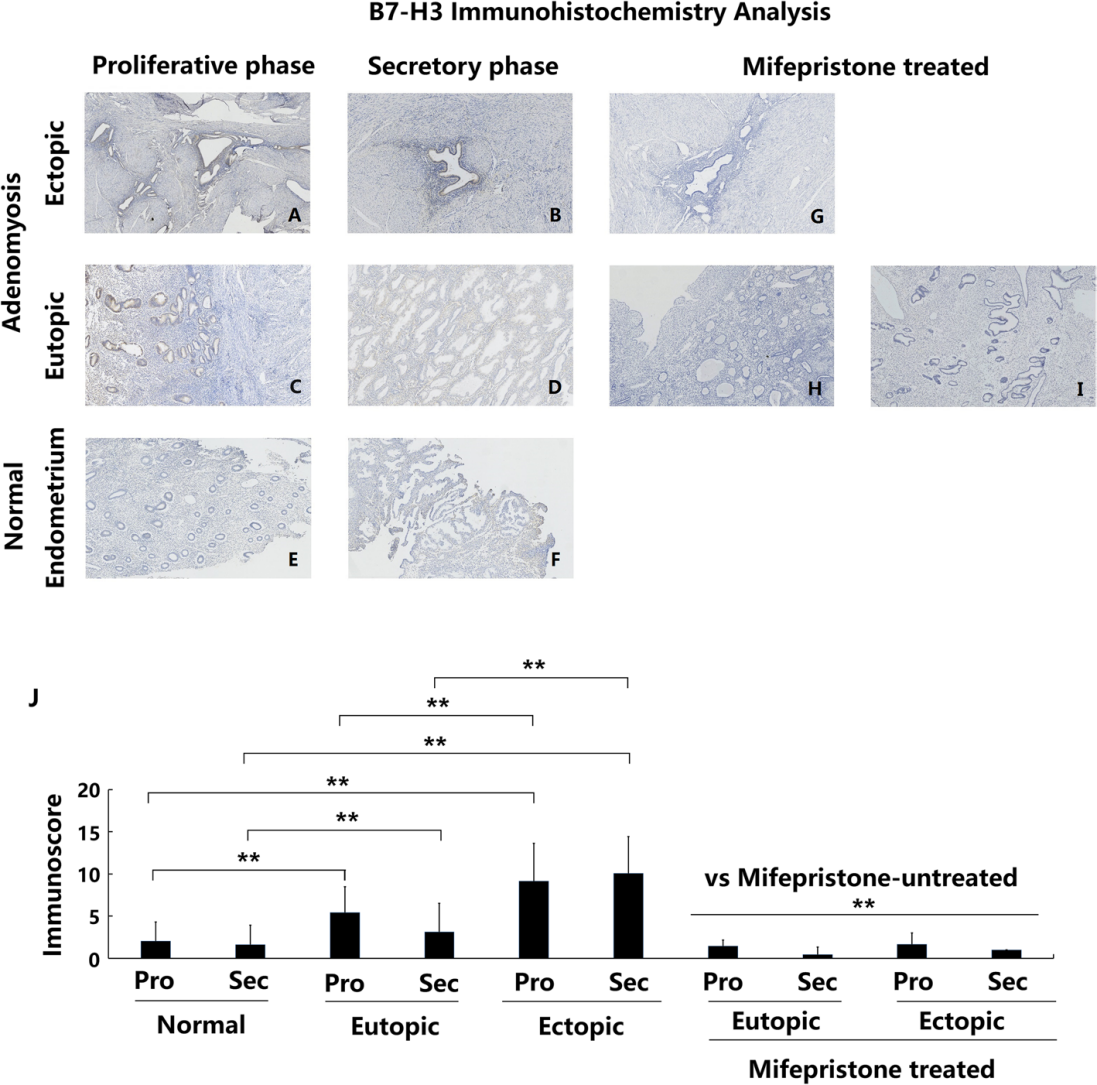
Immunoexpression and comparison of B7-H3 in normal, eutopic and ectopic endometrium of adenomyosis treated with and without mifepristone. **a** Ectopic endometrium of proliferative phase in patient with untreated adenomyosis (*n* = 35); **b** Ectopic endometrium of secretory phase in patient with untreated adenomyosis (*n* = 23); **c** Eutopic endometrium of proliferative phase in patient with untreated adenomyosis (*n* = 35); **d** Eutopic endometrium of secretory phase in patient with untreated adenomyosis (*n* = 23); **e** Normal endometrium of proliferative phase in patients without adenomyosis (*n* = 47); **f** Normal endometrium of secretory phase in patients without adenomyosis (*n* = 27); **g** Ectopic endometrium in patient with mifepristone-treated adenomyosis (*n* = 11); **h** and **i** eutopic endometrium in patient with mifepristone-treated adenomyosis (*n* = 11); **j** Immunoscore comparation of B7-H3 between each groups, * *P* < 0.05, ** *P* < 0.01. a- i magnification: × 100

The original article [1] has been updated.
